# Risk Associated with the Release of *Wolbachia*-Infected *Aedes aegypti* Mosquitoes into the Environment in an Effort to Control Dengue

**DOI:** 10.3389/fpubh.2016.00043

**Published:** 2016-03-22

**Authors:** Justine V. Murray, Cassie C. Jansen, Paul De Barro

**Affiliations:** ^1^CSIRO, Brisbane, QLD, Australia; ^2^Metro North Public Health Unit, Queensland Health, Brisbane, QLD, Australia

**Keywords:** impact assessment, dengue, *Wolbachia*, *Aedes aegypti*, release, risk analysis

## Abstract

**Background:**

In an effort to eliminate dengue, a successful technology was developed with the stable introduction of the obligate intracellular bacteria *Wolbachia pipientis* into the mosquito *Aedes aegypti* to reduce its ability to transmit dengue fever due to life shortening and inhibition of viral replication effects. An analysis of risk was required before considering release of the modified mosquito into the environment.

**Methods:**

Expert knowledge and a risk assessment framework were used to identify risk associated with the release of the modified mosquito. Individual and group expert elicitation was performed to identify potential hazards. A Bayesian network (BN) was developed to capture the relationship between hazards and the likelihood of events occurring. Risk was calculated from the expert likelihood estimates populating the BN and the consequence estimates elicited from experts.

**Results:**

The risk model for “Don’t Achieve Release” provided an estimated 46% likelihood that the release would not occur by a nominated time but generated an overall risk rating of very low. The ability to obtain compliance had the greatest influence on the likelihood of release occurring. The risk model for “Cause More Harm” provided a 12.5% likelihood that more harm would result from the release, but the overall risk was considered negligible. The efficacy of mosquito management had the most influence, with the perception that the threat of dengue fever had been eliminated, resulting in less household mosquito control, and was scored as the highest ranked individual hazard (albeit low risk).

**Conclusions:**

The risk analysis was designed to incorporate the interacting complexity of hazards that may affect the release of the technology into the environment. The risk analysis was a small, but important, implementation phase in the success of this innovative research introducing a new technology to combat dengue transmission in the environment.

## Introduction

Dengue remains a priority for public health authorities across the globe. The viral disease is transmitted primarily by the mosquito *Aedes aegypti* (*Ae. aegypti*), which also transmits a number of other viruses, including yellow fever and chikungunya viruses (1, 2). An estimated 390 million dengue infections occur annually as the virus expands into new geographic regions and affects both urban and rural settings (3, 4). Dengue is not considered endemic in Australia, and dengue activity is currently limited to some parts of Queensland, where *Ae. aegypti* is present (5, 6). In the absence of a vaccine, management of dengue comprises vector management and reduction of human exposure to mosquito bites through the use of repellents and behavioral modification (7). However, outbreaks still occur as a result of the repeated reintroduction of the virus through infected overseas travelers visiting or returning to Australia, especially during summer months.

*Aedes aegypti* females are responsible for transmitting dengue viruses between humans. However, the mosquito must first acquire the virus from a viremic individual through a blood meal. While *Ae. aegypti* is African in origin, it is now distributed in many tropical and subtropical regions across the globe. It is almost always associated with human habitats. Larval development most commonly occurs in artificial water containers, such as pot plant bases, discarded tires, and water tanks (8, 9), or natural containers, such as fallen palm fronds and coconut husks. The potential for larval production can be high, especially within urban centers of developing countries, where domestic water storage in containers is common (10, 11).

*Wolbachia* are obligate intracellular endosymbiotic bacteria found naturally in a wide range of invertebrates, including some species of mosquitoes, but not *Ae. aegypti* (12, 13). *Wolbachia* are maternally transmitted to the next generation through the eggs, infect reproductive tissues, and manipulate the host reproductive cycle to increase their spread (14–16). Reproductive strategies associated with *Wolbachia* infection include parthenogenesis, male killing or feminization, sex-ratio distortions (17–19), and cytoplasmic incompatibility (20, 21), which effectively reduce the ability for the dengue virus to infect other hosts by blocking virus replication (2). Many years of field-based and laboratory studies have resulted in the successful introduction of at least two *Wolbachia* strains (*w*Mel and *w*MelPop-CLA strain) to *Ae. aegypti* populations [see Ref. (22) for details].

As part of a larger international project to reduce the incidence of dengue fever in Australia and elsewhere around the world, a trial field release of *Wolbachia*-infected *Ae. aegypti* was proposed for the 2010/11 wet season in far North Queensland, Australia. The biosafety of proposed release was assessed and approved by the Australian Government (23). This was the first time such a release had been considered and a key element in securing permission was the analysis of risks associated with the release. Thus, all elements of risk associated with the release of *Ae. aegypti* containing *Wolbachia* into naturally occurring populations needed to be identified and investigated before the release was approved to ensure that the field release would “cause no more harm” than that posed by natural *Ae. aegypti* populations. The novelty of the project meant that there were limited empirical data and subsequently high levels of uncertainty surrounding the potential for negative impacts. Where historical data relevant to assessing risk are lacking, elicitation of expert knowledge is an appropriate proxy for empirical data and is often used to address uncertainties in knowledge assumptions and limited datasets (24). Because of the novelty of the system, and potentially wide-ranging effects on individuals and communities in release areas, and in order to ensure transparency in the process, non-technical community experts were involved in the elicitation of risk estimates as well as technical experts.

Risk assessments are standard practice in many business practices and operational procedures of organizations today. A risk analysis determines the likelihood of an event occurring and the consequences of an event if it does occur. The level of risk is calculated from the product of the likelihood and consequence. A risk assessment needs measures to incorporate feedback opportunities to improve predictions and reduce uncertainty (25, 26).

The results of the risk analysis described herein were used to determine the approval of the proposed release. To assess the risk associated with the proposed release, two risk end points (undesirable states of a system) were considered. Here, we describe the tools, process, and methodology used in the risk analysis to identify and assess potential hazards of releasing *Ae. aegypti* mosquitoes containing a strain of *Wolbachia* into a naturally occurring population. We also describe the results of the risk analysis, including discussion of the specific hazard domains identified, the Bayesian networks (BNs) constructed to explore the hierarchal structure and relationship of the hazards, and the final expert-derived estimates of risk associated with the release of the modified *Ae. aegypti* in Australia. In addition, we list recommendations for developing risk analyses, which address novel technologies with diverse impacts.

## Materials and Methods

### Steps of Risk Analysis – Overall Methodology

The risk analysis for the proposed release assessed the risks associated with two end points: (1) that release would not occur within a set time frame due to logistical, regulatory, political, epidemiological, and community concerns, referred to as “Don’t Achieve Release” (DAR) and (2) that the release of the modified *Ae. aegypti* would result in more harm through impacts on the economy, social wellbeing and community health, future mosquito control effort, and/or adverse changes to the biology of the vector, *Wolbachia* or dengue viruses, in the release locations when compared with the current situation within a 30-year timeframe, known as “Cause More Harm” (CMH) (Table 1).

**Table 1:** **Definition of key nodes and states for the two endpoints produced from expert workshop**.

Endpoint	Node	Definition
Don’t Achieve Release	Logistical constraints	Restrictions in achieving release due to insufficient numbers of mosquitoes to release, insufficient funding to support activities, unfit biological traits in mosquitoes, and/or an unfavorable release site caused by it not being suitable as a physical environment or because of epidemiological issues
	Compliance	Inability to comply because of political (incl. adverse or no media coverage) and/or community opposition with the community not engaged, formal regulatory oversight, and other oversight
	Public opinion	Non-acceptance of release because of political (incl. adverse or no media coverage) and/or community opposition with the community not engaged
Cause More Harm	Standard of public health	Release results in increased health issues by increasing dengue transmission, increasing densities or frequency of biting (nuisance biting), or increasing likelihood of transmitting other pathogens
	Avoidance strategies	A change in normal public behavior to avoid contact with *Ae. aegypti* by increasing avoidance behavior and insecticide use or removing breeding sites around dwelling
	Mosquito management efficacy	Reduction of the effectiveness of, or increased requirement for, mosquito control due to insufficient monitoring of any change in mosquito numbers, increased insecticide resistance, and a need to apply more or greater diversity of treatments
	Ecology	Ecological harm resulting from the release due to transfer of *Wolbachia* to another invertebrate or vertebrate species or an increase in the mosquito’s geographic range through climate tolerance or changes in host range, a broadened or changed ecological niche, or an increased density of mosquitoes
	Economic effects	Economic harm attributable to the release through an increased costs of health care, reduction in tourist numbers, decreased property values, and/or reducing supply of workers or increasing costs for employers

*Other node definitions are listed in Table S1 in Supplementary Material*.

Following the formulation of the two end points, DAR and CMH, risk was assessed through four iterative stages: (i) hazard identification and development of a conceptual model, (ii) development of a predictive risk model, (iii) model scrutiny and update, and (iv) risk calculation. These stages were undertaken using various methods, including expert elicitation *via* workshops and email correspondence, and construction of a BN. A series of expert workshops were held in Cairns and Brisbane, QLD, Australia, in 2010. Each workshop is comprised a range of “experts” as participants, including academics, regulatory officials, and community members, to obtain the broadest knowledge possible. Technical experts were identified due to their association with the project or as local experts with relevant expertise on mosquitoes, vector control, arboviruses, and public health. Non-technical community experts and non-government organizations were identified through a community engagement program that was conducted in the proposed location of release (27).

The authors facilitated each workshop using an elicitation method adapted from Spetzler and von Holstein (28) and O’Hagan et al. (29) using breakout groups of three to four experts to encourage individual expert input, combined with entire group participation for feedback opportunity and group consensus. Averaging expert responses through group consensus counters individual variation in opinion (30) but does not account for outlier influence. Therefore, it took email correspondence and a further two workshops to reach consensus across each model.

### Hazard Identification and Development of the Conceptual Model

The first step in any risk analysis is to identify all hazards associated with an event. An initial workshop was conducted with technical experts to describe all potential risks or hazards that may result from the proposed release relevant to each endpoint. The initial step elicited expert judgment from researchers associated with the Grand Challenges in Global Health (GCGH) initiative during a 2-h brain storming and hazard mapping session on May 20, 2009. Participants were provided the two end points and asked to describe relevant types of potential hazard under general categories before identifying specific hazards for each. Second, additional hazards of concern to the proposed release locations were elicited through the GCGH’s public engagement program through community seminars and media (27, 31).

### Development of the Predictive Risk Model

A BN was used as the model structure to determine the risk of the end points using the information derived from the experts. A BN is an influence diagram that depicts logical or causal relationships of factors that can influence the likelihood of an outcome of a parameter (32). The BN influence diagram consists of a graph (network) with a set of connected nodes (representing hazards), where directed connections from “parent” nodes leading to a “child” node indicate a causal influence of the parent node(s) on the child node. Each node is discretized into categories (states) defining the range or value of information represented in each state for each node (33). Underlying each child node is a conditional probability table (CPT) that defines the dependencies between the parent nodes and their associated states (34). The likelihood of each state for each child node is updated when values for a parent node are specified (by expert estimates or empirical data). We used Netica 4.12 (35) to develop and compile the BN.

Experts were invited to critique the model structure of the BN and edit accordingly by adding, removing, or modifying nodes (hazards). Experts also formulated the definitions for the nodes and their associated states. Once the model structure was agreed upon, experts were asked to estimate the conditional probabilities for each combination of states possible under each child node. Subsequent discussion by email and two further workshops with experts resulted in consensus for the final BN models and underlying CPTs.

### Model Analysis and Sensitivity Analysis

We ran sensitivity analysis within Netica 4.12 to examine the influence of each node on the two end points. Sensitivity analysis in Netica uses entropy reduction (the expected decrease in uncertainty of the node being queried due to information at the parent node) to determine how “sensitive” a model is to the changes in model variables (36). Hence, if prior findings or “priors,” such as knowledge from an expert’s experience, are supplied about the state of a parent node, this may reduce the maximum range of uncertainty around the likelihood distribution of the output child node (35, 37). Sensitivity analysis can also show how influential each parent node is toward its associated child node. This is determined by the likelihoods given in the CPTs populated by the experts.

Uncertainty was reduced by continually incorporating new information from emerging research as “findings” when it became available, approaching all potential experts for participation, educating all participating experts regarding the model language, jointly developing definitions for each of the model nodes and associated states, and having an independent committee overseeing each stage of the risk analysis. Email correspondence and further workshops with technical experts were conducted to address a number of issues, including reducing the uncertainty around nodes by narrowing their expected distributions (e.g., uncertainty range changes from 0.1–0.9 to 0.4–0.6 when new knowledge reduced the uncertainty around a variable). In the final workshop, experts were asked to come to a group consensus through discussion of the merits of the current individual likelihoods for each node and adjusting where necessary, thus reducing the uncertainty through group experience.

### Risk Calculation

Risk was calculated by combining the probability or likelihood of an event occurring (or not occurring) and the impact or consequences of that event occurring (or not occurring). Hence, we considered the likelihood of not achieving release and that the event will cause more harm, as well as the consequences if these events occurred. Estimates for both likelihood and consequence were elicited from the technical experts for calculation of risk. These estimates were multiplied together to give a risk value. A scale was determined with experts to rank risk from negligible to very high (Table 2). These were combined into a risk matrix, which corresponded with the risk value.

**Table 2:** **Scale for risk used for calculating the risk associated with the two endpoints**.

Scale	Negligible	Very low	Low	Moderate	High	Very high
Probability	0–0.01	0.02–0.10	0.11–0.30	0.31–0.74	0.75–0.94	0.95–1.0

*Risk was calculated as a product of the likelihood of an event happening and the consequence of an event happening*.

## Results

### Hazard Identification and Development of the Conceptual Model

Fifty-two possible hazards associated with the release of the modified *Ae. aegypti* were initially identified at the first GCGH session across 13 categories, including regulatory compliance, community acceptance, and adverse public health effects, beyond that already caused by naturally occurring *Ae. aegypti*. The expert workshops further identified additional hazards. After workshops, consultations and community engagement, a total of 109 hazards were identified and defined (Supplementary Material). After removing hazards that were beyond the scope of the end points, the remaining hazards were refined by grouping similar hazards into themes: (i) logistics, compliance, and public opinion for DAR (comprising 18 nodes) and (ii) ecological impact, economic impact, mosquito management, social behavior changes, and public health impacts for CMH (comprising 30 nodes).

### Predictive Risk Model

The hazard themes provided a BN framework for the endpoints, which was critiqued and modified by experts to best reflect causal relationships of each hazard (defined in Table 1). Experts estimated the likelihoods to populate each parent node. The CPTs are provided in Tables S1–S20 in Supplementary Material.

**Figure 1:**
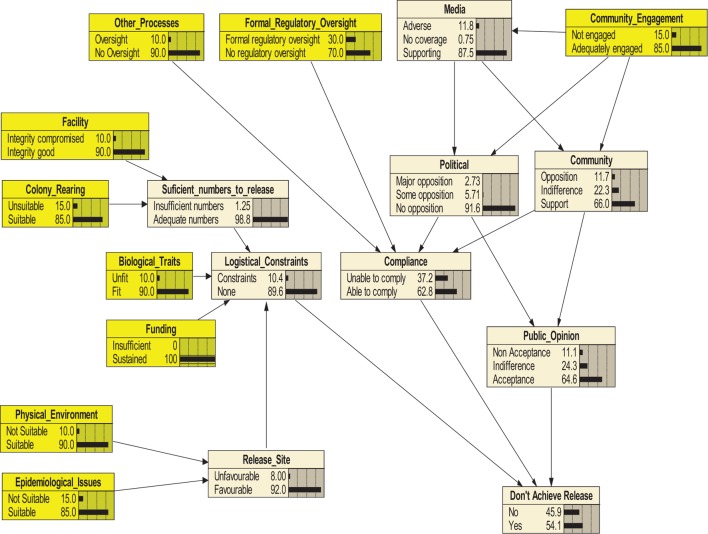
**Bayesian belief network for the endpoint “Don’t Achieve Release.”** Each node (box) and the states within nodes are described in Table 1 and Table S1 in Supplementary Material. Probabilities for terminal nodes are determined by expert estimates. Parent nodes are represented in yellow.

**Figure 2:**
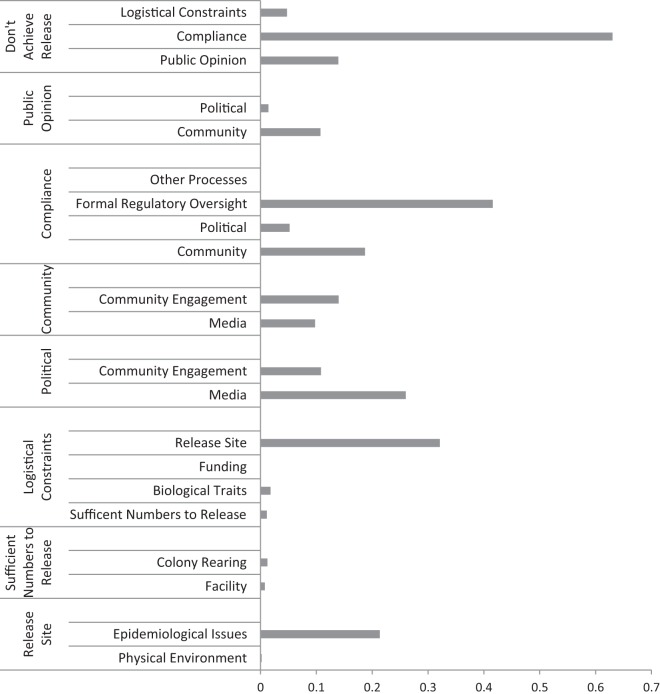
**Sensitivity results (measured as entropy reduction) for the Bayesian belief network for the “Don’t Achieve Release” endpoint**. It shows the sensitivity of the key variables (logistical constraints, compliance, and public opinion) along with the sensitivity of the variables inputting these and of the “Don’t Achieve Release” output. The longer the bar length, the more influence the variable had on the model within each category.

Overall, the conditional probability that the release would not occur on time due to a hazard failure was estimated at 45.9% (moderate likelihood) (Figure 1). Sensitivity analysis revealed DAR was the most sensitive to the “compliance” node (>60%), which was in turn strongly influenced by the “formal regulatory oversight” (45%) and to a lesser extent the “community” (20%) and “political” (7%) nodes (Figure 2). “Public opinion” (15%) was sensitive to the “community,” and the “logistical constraints” node was sensitive to the “release site,” which was in turn influenced by “epidemiological issues.”

**Figure 3:**
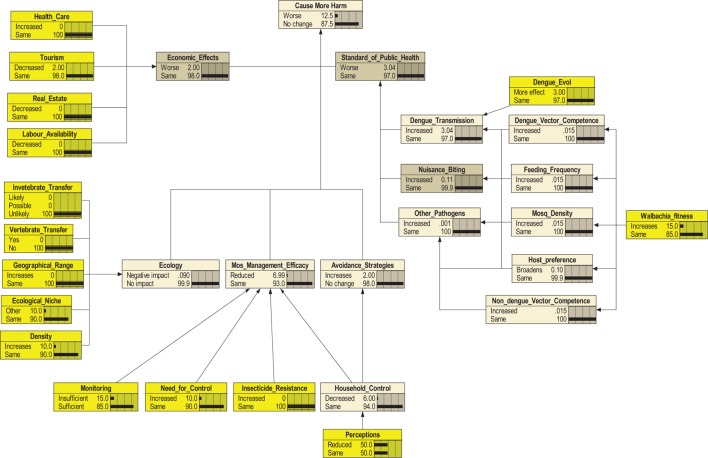
**Bayesian belief network for the endpoint “Cause More Harm.”** Each node (box) and the states within nodes are described in Table 1 and Table S1 in Supplementary Material. Probabilities for terminal nodes are determined by experts. Parent nodes are represented in yellow.

**Figure 4:**
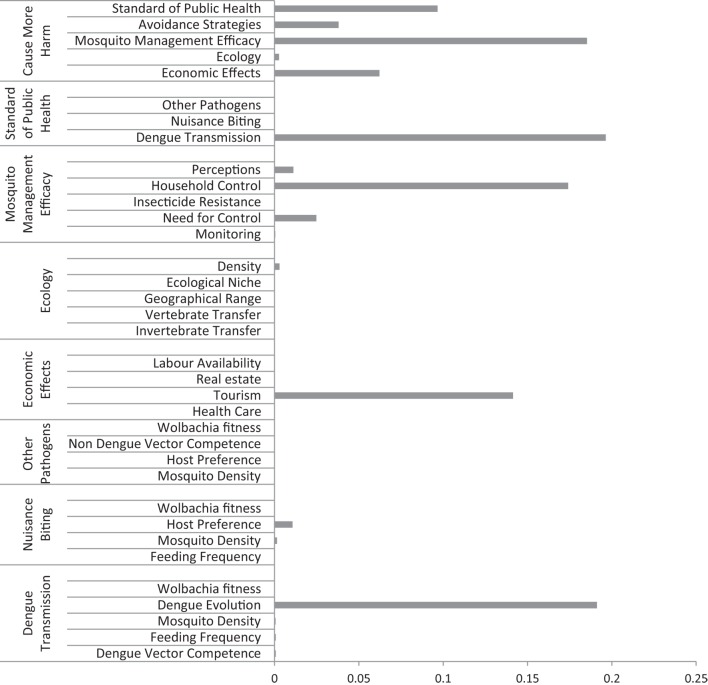
**Sensitivity results (measured as entropy reduction) for the Bayesian belief network for the “Cause More Harm” endpoint**. It shows the sensitivity of the key variables (standard of public health, avoidance strategies, mosquito management efficacy, ecology, and economic effects) along with the sensitivity of the variables inputting these and of the “Cause More Harm” output. The longer the bar length, the more influence the variable had on the model within each category.

The probability that some forms of additional harm could eventuate over a 30-year time frame from the date of release was 12.5% (Figure 3). Sensitivity analysis (Figure 4) revealed CMH was most sensitive to the “efficacy of mosquito management” (18%), which in turn was influenced strongly by “household control.” “Standard of public health” (10%) had some influence, followed by “economic effects” (6%) and “avoidance strategies” (4%) nodes. The node “Ecology” had little effect. “Standard of public health” was strongly affected by “dengue transmission,” which in turn was heavily influenced by “dengue evolution.” Change in “tourism” had the strongest effect on the “economic effects” node (Figure 4).

### Model Updating

Owing to the technical nature of the research and the use of a range of experts from different backgrounds and their varied knowledge of the project, different types and levels of uncertainty were evident (Murray et al., in preparation). Uncertainty arose from a number of sources, including linguistic interpretation of the hazard definitions (Table 1). Uncertainty was particularly apparent around knowledge gaps and when experts were unfamiliar with terminology, techniques, and the modeling process. Uncertainty was typically evident by a broad distribution between low and high values in elicited likelihoods (e.g., event will occur between the range of 0.1 and 0.9 likelihood). Reducing uncertainty narrows the range of possible likelihoods. Throughout the workshops and correspondence, experts were continually encouraged to provide updates on any new data arising from research currently underway and work toward consensus and removing any divergence and outliers. Effort was also invested in educating experts to reach a common linguistic understanding of node definitions. Models were continually updated when consensus was reached or new knowledge became available. For example, new information was released to reduce the uncertainty around infecting non-target species and feeding on other hosts (38).

### Risk Calculation

Consensus of expert opinion on the endpoint for DAR resulted in a low likelihood (0.20) and a moderate consequence (0.35), ensuing DAR to be of very low risk (Table 3). “Biological traits” that were not suitable, a compromised “facility” and “insufficient numbers to release,” had high consequence but a very low likelihood, resulting in very low risk. This was the same level of risk for “insufficient funding,” which had a very high consequence but negligible likelihood. “Public opinion,” “community compliance,” “political,” “media,” “physical environment,” and “other processes” had negligible risk. The remaining nodes had very low risk except for problems encountered with “colony rearing,” which had a high consequence and low likelihood resulting in low risk (Table 3).

**Table 3:** **Risk matrix depicting the risk associated with Don’t Achieve Release**.

		Consequence						
		Negligible	Very low	Low	Moderate	High	Very high
**Likelihood**	**Negligible**	Negligible risk	Negligible risk	Negligible risk	Negligible risk	Negligible risk	Very low risk Funding
	**Very low**	Negligible risk	Negligible risk Public opinion	Negligible risk Other processes Political	Negligible risk Media Physical environment	Very low risk Biological traits Sufficient numbers to release Facility	Low risk
	**Low**	Negligible risk	Negligible risk	Negligible risk Community compliance	Very low risk Community engagement *Don’t Achieve Release* Formal regulatory oversight Logistical constraints Release site Epidemiological issues	Low risk Colony rearing	Moderate risk
	**Moderate**	Negligible risk	Negligible risk	Very low risk	Low risk	Moderate risk	High risk
	**High**	Negligible risk	Very low risk	Low risk	Moderate risk	High risk	Extreme risk
	**Very high**	Negligible risk	Very low risk	Low risk	Moderate risk	High risk	Extreme risk

*Risk was calculated as the product of likelihood × consequence and ranked according to the categories in Table 2*.

Consensus of expert opinion on the endpoint for CMH resulted in a very low likelihood (0.10) and a very low consequence (0.10), ensuing CMH to be of negligible risk (Table 4). Community “perceptions” had the highest risk with moderate likelihood (0.50) and moderate consequences (0.4) combined to render low risk. Community “perceptions” directly affected “household control.” However, “household control,” along with “avoidance strategies,” “mosquito density,” and “*Wolbachia* fitness,” had low likelihood and moderate consequence leading to very low risk. The remaining nodes relevant to CMH had negligible associated risk (Table 4).

**Table 4:** **Risk matrix depicting the risk associated with Cause More Harm**.

		Consequence						
		Negligible	Very low	Low	Moderate	High	Very high
**Likelihood**	**Negligible**	Negligible risk Dengue vector competence Vertebrate transmission	Negligible risk Real estate Standard of public health	Negligible risk	Negligible risk Ecology Geographical range Health care Host preference Insecticide resistance Invertebrate transfer	Negligible risk Labor availability	Very low risk
	**Very low**	Negligible risk	Negligible risk *Cause More Harm* Dengue transmission Non-dengue vector competence Other pathogens Tourism	Negligible risk Dengue evolution Economic effects Nuisance biting	Negligible risk Density Ecological niche Feeding frequency Mosquito management efficacy Need for control	Very low risk	Low risk
	**Low**	Negligible risk	Negligible risk	Negligible risk Monitoring	Very low risk Avoidance strategies Household control Mosquito density *Wolbachia* fitness	Low risk	Moderate risk
	**Moderate**	Negligible risk	Negligible risk	Very low risk	Low risk Perceptions	Moderate risk	High risk
	**High**	Negligible risk	Very low risk	Low risk	Moderate risk	High risk	Extreme risk
	**Very high**	Negligible risk	Very low risk	Low risk	Moderate risk	High risk	Extreme risk

*Risk was calculated as the product of likelihood × consequence and ranked according to the categories in Table 2*.

## Discussion

### Overall Risk

Managing risk is an important component in most research and management endeavors. A risk analysis was undertaken on the proposal to release *Wolbachia*-infected *Ae. aegypti* in North Queensland, Australia, to reduce the transmission of dengue. Risk analysis considers the adverse potential outcomes that could eventuate from an event. Thus, we were only interested in whether release was not achieved and whether additional harm may result from this process. The risk assessment revealed that there was a higher likelihood of the project not achieving release than causing more harm, although the risk for both were very low. The potential risk for CMH was primarily influenced by the efficacy of alternative mosquito management actions, and the potential risk of DAR was mainly affected by whether regulatory compliance was obtained.

### Don’t Achieve Release

Regulatory compliance, either formal or informal, was considered a hazard because of potential delay in the approval for the release and, hence, the release not being achieved within the designated timeframe. Compliance, in turn, was affected by the lack of formal regulatory oversight. The novelty of the proposed release was reflected in the initial failure to identify an appropriate regulatory body, which would accept governance over the proposed release and prescribe an appropriate risk analysis framework. Genetically modified organisms (GMOs) are controlled by regulatory structures (39). Notably, the modified *Ae. aegypti* was determined not to be a GMO by the relevant organization [The Office of the Gene Technology Regulator (OGTR)] on the basis that the method of modification did not involve recombinant technology. Nonetheless, the decision was made to use the OGTR risk analysis framework for GMOs (40) as best practice in the absence of regulatory guidance. In addition, an independent panel of local and international experts in risk analysis, biological control, and regulation were convened to oversee the methods used. The complete risk analysis was made publicly available and used to support the search for a regulator. This is an unusual situation as the risk analysis was completed before a regulator, and their requirements had been identified. Furthermore, an unusual regulatory solution was eventually found with the consideration of *Wolbachia* as a veterinary chemical substance governed under existing legislation (23).

The risk that public opinion may prevent release was also considered influential as it was difficult to preempt the community response to the project, and community engagement was considered vital for project success. To address this risk, long-term research had been conducted using an engagement strategy and communication materials specific to the local sociopolitical context for the communities at the release sites. Information sessions were run for the community, discussing past control methods for dengue and their limitations, the fundamentals of the project, and the expectations regarding outcomes of the project. The communities were involved and their concerns addressed at every step, enabling a reduction in the risk of opposition to the project (27, 31).

### Cause More Harm

Causing more harm was considered a negligible risk. The greatest concern was whether general “mosquito management efficacy” would be maintained or if it was potentially reduced by households decreasing their efforts of mosquito control. As a consequence of the local population considering the threat of dengue transmission to be reduced following the release of *w Ae. aegypti*, residents may decrease efforts to minimize mosquito breeding around their home. This concern highlights the potential need for more effort and education directed toward the community to ensure that ongoing mosquito management is maintained at the household level (41, 42). Cliff and Campbell (43) also considered it was important to include perceptions, particularly behavioral intent and concern, within a biosecurity risk assessment to provide a more effective and efficient understanding of risk.

The standard of public health was also of some concern due to the potential risk of the release causing possible evolution of the dengue viruses to become a greater threat to public health. However, the likelihood of this occurring was deemed low by the technical experts. While Bennett et al. (44) suggest that dengue virus evolution can occur rapidly, the experts agreed that there is little evidence that the presence of *Wolbachia* in the mosquito will increase this risk, especially as evolutionary dynamics can only occur when the virus is transmitted successfully. In this case, the *Wolbachia* infection itself reduces the likelihood that transmission will occur (22).

### Expert Elicitation

Releasing mosquitoes into the environment, in this case around human habitation, affects local communities where the release sites are targeted. This necessitates the involvement of the community through the whole process, from providing information sessions and literature (27) to inclusion in expert panels. Community experts were able to provide valuable contribution to assessing the likelihoods describing the economic effects and avoidance strategies for CMH. Community experts were also encouraged to participate in populating the other nodes but found them to be irrelevant to their personal experiences or difficult to understand the technicalities underlying the other nodes.

Because of the novelty of the technique, scientific experts were needed to populate technical nodes, such as ecology, dengue transmission, and *Wolbachia* fitness. The complexity of the model required a number of workshops to obtain full understanding and consensus for the different technical components of the model, especially those describing dengue evolution and *Wolbachia* fitness.

### Risk Analysis Methodology

The four stages of the risk analysis were necessary to accommodate the complexity of the events being modeled and the uncertainty that arose from considering such innovative techniques (Murray et al., in preparation). This stepwise process provided an opportunity to collect feedback on model structure and relationships and reduce the uncertainty around nodes captured in the risk model. The brainstorming enabled all hazards to be identified and be sorted into appropriate themes directed toward each negative endpoint. Once hazards were identified and the model structure determined, BNs provided opportunity for recording likelihoods of events. It was important to minimize unnecessary complexity and keep models simple, wherever possible (45). Careful editing of models can include hazard grouping to represent overall relationships and removing rare or irrelevant hazards to allow streamlining of the model structure. The BNs can capture complex interactions between different hazards in a simplified and intuitive way. When involving non-technical community experts, it is especially important to provide them with a platform that encourages understanding with limited prior knowledge.

When estimating risk, capturing the likelihoods of an event is only half the story. An event may have a high likelihood of occurring but the consequences of that event may be minimal. Hence, combining the BN likelihood results with the associated consequences within a matrix provided a realistic estimate of the risk associated with that event (46–48). For example, “community perceptions” of dengue control had the highest risk for CMH, but this was still considered a low risk due to a minimal consequence of this outcome.

### Limitations

In this assessment, expert’s opinion was used to provide likelihoods of hazard failures as a surrogate for incomplete or absent data, but this approach has limitations. For example, expert judgment is based on observation and experience, which varies both between and within the research and community representatives (49). How individuals perceive and quantify numerical risk also varies (50). Uncertainty was evident in different forms [including variability and epistemic and linguistic uncertainty (29, 51)] and although steps were taken to minimize its effect, in some cases, these efforts may have enhanced it. For example, the hazard definitions were intended to be succinct and accessible to both science and community representatives and included a glossary of key terms to avoid vagueness (Table 1; Supplementary Material). However, the elicitation exercise sent *via* email was notable for the linguistic difficulty some respondents experienced regarding the definitions – particularly if they had not participated in the earlier stages of model development. This manifested as highly divergent hazard scoring (broad ranges or outliers) and in some cases a lack of confidence in assigning a likelihood estimate to a hazard. The inability to easily discuss aspects, such as definitions and reach consensus on interpretation, and the occasionally low response rates are failings of this individual-focused remote approach (52). However, the value of an individual approach lies in the fact that a set of estimates from prior knowledge (known as “*priors*”) can be rapidly obtained without group influence. Individual priors may represent more breadth of knowledge than priors determined through group consensus. The last expert workshop was designed in response to these issues with an aim to reduce uncertainty and obtain a consensus set of *priors*, which adequately reflected individual expert opinion. The *priors* also represent a baseline for examination of the likelihoods of hazard failure and the consequences of failure. These *priors* can be updated when new information becomes available to further inform expert estimates or when new data can be used to replace expert estimates. For instance, where robust scientific data from laboratory testing are available on horizontal transfer rates, it may be more accurate than the equivalent expert opinion.

Risk matrices have been used extensively in risk analysis and management but suffer some limitations. Cox (53) notes that risk matrices can have poor resolution by only comparing a fraction of hazard pairs, can erroneously assign higher risks to qualitative rather than quantitative risks, and can lead to ambiguous inputs and outputs if consequences are uncertain. During our risk analysis process, we endeavored to counter these limitations. Using BNs allowed the modeling of interactions between all associated risks so all hazards, and their potential codependent hazards are accounted for within the risk matrix. The BNs were also populated entirely by expert elicitation; therefore, quantitative data were not used. However, we continually sought feedback from experts to ensure that the latest knowledge and understanding was captured in the models. Lastly, we chose technical experts with great expertise within the field to quantify consequences both individually and as a group.

While it is difficult to test and validate risk assessment models, the model results informed the independent scientific committee and the regulatory body who subsequently approved the project. Post-project results showed that the project members were able to overcome any potential risks for DAR by successfully producing the modified *Ae. aegypti* populations for the designated release timeframe (23). *Wolbachia*-infected mosquito populations to date have also been successfully established into their natural environment around the release sites (54). However, risks associated with CMH need to be monitored over the next 30 years.

## Conclusion

The risk analysis highlighted factors that needed to be addressed before the release of *Wolbachia*-infected mosquitoes could occur. It also highlighted the uncertainty that arises from innovative research and the employment of expert opinion to populate models. We make the following five recommendations:

- Overall community engagement and media coverage need to play a significant role in obtaining project approval, especially if there are direct implications to the community.
- Inclusion of both technical and non-technical community experts captures the diversity of opinions relevant to different hazards. This is especially important when community support is essential.
- An iterative model continually updated with new knowledge can help reduce the uncertainty associated with risks as well as highlight where new research is needed to clarify issues.
- As in this case, innovative research may not be covered by existing regulatory bodies and this may lead to delays. Understanding this early can provide an opportunity and time for project managers to find solutions.
- Understanding the role that individual and community perceptions play when introducing new technology for which there is no prior example of application is very important, particularly when modeling future human behavior. In this case, introducing this technology may have led to individuals believing the dengue threat had been alleviated and thus reducing the amount of household mosquito control they conduct. Thus, adopting ethical public participation and engagement sessions to both inform and answer direct questions from members of the community can reduce this risk (27, 42, 55).

## Ethics

Human research ethics was approved through the Human Research Ethics Committee (James Cook University) Approval H3555, modifying mosquito population age structure to eliminate dengue transmission: Part 1 population attributes of the dengue vector and Part 2 social research and community engagement. Institutional Biosafety was approved through the University of Queensland Institutional Biosafety Committee Approval IBC/Biosafety17/SBS/2010, field testing of *Wolbachia Aedes aegypti* to control dengue transmission in North Queensland.

## Author Notes

JM is an ecologist with extensive experience in expert elicitation and BNs. CJ is an entomologist with extensive knowledge in mosquito research and vector-borne diseases. PDB is the Research Director of the CSIRO Health and Biosecurity Business Unit’s Risk Evaluation and Preparedness Programme with a broad knowledge in many areas, including vector ecology associated with human health and risk analysis.

## Author Contributions

PDB conceived the idea and chose the methodology for the study. All authors took part in running the expert workshops. JM and CJ analyzed the model data and JM interpreted the results. All authors contributed to the writing of the manuscript.

## Conflict of Interest Statement

Dr. JM reports a grant from the University of Queensland. CSIRO was contracted by the University of Queensland to undertake an independent risk assessment of the proposed release of *Wolbachia Aedes aegypti*. The *Wolbachia Aedes aegypti* research was funded by the Foundation for the National Institutes of Health through the Grand Challenges in Global Health Initiative of the Bill & Melinda Gates Foundation as part of Eliminate Dengue.

## Supplementary Material

A glossary of terms, the definitions of the nodes and states for each end point, the conditional probability tables, and the complete fault trees for the two endpoints can be found at the CSIRO data access portal (http://doi.org/10.4225/08/56806A85E3E65) (56). The email elicitation template is also provided there as Supplementary Information.

## Abbreviations

BN, Bayesian network; CMH, Cause More Harm; CPT, conditional probability table; DAR, Don’t Achieve Release; GCGH, Grand Challenges in Global Health; GMO, genetically modified organism; OGTR, Office of the Gene Technology Regulator.
